# The Association of Peak Glycemia and No‐Reflow Phenomenon in Patients Undergoing Primary Percutaneous Coronary Intervention

**DOI:** 10.1155/crp/9919861

**Published:** 2026-02-13

**Authors:** Golrokh Ghaffari, Maryam Mehrpooya, Mohsen Faghihinia, Elnaz Shahmohamadi, Farnoosh Larti, Babak Geraiely

**Affiliations:** ^1^ Cardiology Department, Imam Khomeini Hospital Complex, Tehran University of Medical Sciences, Tehran, Iran, tums.ac.ir; ^2^ Center for Heart Rhythm Disorders, University of Adelaide, Adelaide, Australia, adelaide.edu.au

**Keywords:** clinical outcomes, no-reflow phenomenon, peak glycemia, primary percutaneous coronary intervention, ST-elevation myocardial infarction, SYNTAX score

## Abstract

**Objective:**

This study investigates the effect of peak glycemia on the no‐reflow phenomenon in patients with ST‐elevation myocardial infarction (STEMI) undergoing primary percutaneous coronary intervention (PPCI) to examine the relationship between elevated blood glucose (BG) levels and no‐reflow.

**Method:**

A total of 252 STEMI patients (81.7% male) who underwent PPCI were enrolled. BG was measured by a glucometer every 6 h for 24 h, starting at the time of patient admission. The maximum measured BG was considered the peak glycemic level. A corrected TIMI frame count (CTFC) of less than 27 was used to define the no‐reflow phenomenon in this study.

**Results:**

42.4% of participants experienced no flow, with a significant association between this condition and diabetes mellitus, peak glycemia, smoking history, and elevated LDL cholesterol levels. Specifically, peak glycemia levels above 180 mg/dL independently increased the odds of no‐reflow occurrence (OR = 8.16, 95% CI = 4.1–16.2, *p* < 0.001).

**Conclusion:**

The importance of monitoring BG levels in STEMI patients, as well as the critical role of a multidisciplinary approach, regardless of diabetic status, in mitigating the risk of no‐reflow and improving clinical outcomes, should be highlighted.

## 1. Introduction

The collaborative effort of a specialized team, involving timely ST‐elevation myocardial infarction (STEMI) management, aims to restore full antegrade blood flow to the infarct‐related artery (IRA), ideally achieving a Thrombolysis in Myocardial Infarction (TIMI) Grade 3, indicating optimal perfusion. According to various reports, the incidence of the no‐reflow phenomenon among patients undergoing primary percutaneous coronary intervention (PPCI) varies depending on the definition of no‐reflow, ranging from 32% [1] to 43% [2]. The related mortality rate with no‐reflow also exhibits variability, ranging from 7.4% to 30.3% [3].

Various diagnostic techniques are used to identify the no‐reflow phenomenon, such as angiography, myocardial contrast echocardiography (MCE), and cardiac magnetic resonance imaging (CMR). MCE is considered the gold standard for diagnosing no‐reflow. In contrast, CMR is regarded as the most sensitive and specific method for assessing the severity of no‐reflow [3].

The no‐reflow phenomenon can also be evaluated by analyzing angiographic films. The corrected TIMI frame count (CTFC) provides a quantitative measure of coronary blood flow.

The no‐reflow phenomenon significantly increases the likelihood of severe clinical complications such as death, recurrent myocardial infarction (MI), reduced left ventricular ejection fraction (LVEF), changes in left ventricular structure, dangerous ventricular arrhythmias, heart failure (HF), and even cardiac rupture. In light of these harmful consequences, it is crucial to accurately identify instances of no‐reflow and the associated predictive factors [3]. Hyperglycemia during STEMI is associated with a subsequent reduction in myocardial salvage [4].

This study investigates the relationship between high blood glucose levels and no‐reflow in diabetic and nondiabetic patients undergoing PPCI and identifies contributing factors associated with its occurrence.

## 2. Method

### 2.1. Study Population

The study, conducted from April 2021 to July 2022, focused on​ STEMI patients aged 18 years or older who were admitted to our center and underwent PPCI. Patients who did not consent to the study, those who received medical treatment or urgent coronary artery bypass grafting (CABG) instead of PPCI, and patients who ceased before stent deployment or had incomplete data were excluded from the study. Culprit lesions limited to the posterior descending artery (PDA), posterior left ventricular (PLV) artery, or saphenous vein graft (SVG) were also excluded from the study as they are not specified in the CTFC.

### 2.2. Study Protocol

Demographic data, including age, height, weight, body surface area (BSA), and body mass index (BMI), were collected upon enrollment. Vital signs, including systolic and diastolic blood pressure and heart rate, were recorded at admission. A family history of coronary artery disease was documented according to the American Heart Association (AHA) guidelines [5], and lifestyle factors such as alcohol consumption and smoking history were recorded. Medical histories of Type 2 diabetes, hypertension, hyperlipidemia, obstructive sleep apnea, stroke, and peripheral artery disease were also documented.

A history of diabetes was defined by the American Diabetes Association’s 2022 guidelines using HbA1c and fasting blood glucose cutoffs (HbA1c > 6.5%, FBS > 126 mg/dL) or a self‐report. The history of hyperlipidemia was obtained through self‐report.

CCU nurses measured blood glucose levels at admission and every 6 h for the first 24 h using a blood glucose meter (Clever Chek TD‐4230). They cleaned the index finger pad with an alcohol swab, and then dried it with alcohol. Then, they used a single‐use lancet to draw blood.

The blood was correctly applied to the testing strip, and the reported blood sugar (BS) was documented.

A blood glucose check with the blood glucometer was performed immediately upon admission and every 6 h for the first 24 h, with the results accurately recorded. The highest level detected was reported as peak glycemia.

Initial complete blood samples, drawn from an antecubital or dorsal metacarpal vein, were tested for complete blood count (CBC), C‐reactive protein (CRP), liver enzymes, and kidney function. Also, fasting blood samples were collected on the first day after admission to measure HbA1c and lipid levels.

Experienced operators performed PPCI. Later, the SYNTAX score, TIMI flow, culprit lesions, and the number of diseased vessels were reported. Our study used the CTFC to define the no‐reflow phenomenon.

To objectively assess coronary flow as a continuous quantitative measure, we counted the number of cine frames until contrast initially reached predefined distal coronary landmarks within the IRA, known as the TIMI frame count. The TIMI frame count starts with the first frame in which the contrast dye fully enters the artery. This is determined when three specific criteria are met: (1) a column of nearly fully concentrated dye must span the entire width of the artery’s origin; (2) the dye must contact both edges of the artery’s origin; and (3) the dye must show forward movement. When the left anterior descending artery (LAD) is selectively engaged and the left circumflex artery (LCx) is identified as the problematic vessel, the TIMI frame count begins as soon as the dye first contacts both edges at the LCx’ s origin. The same principle applies when selectively engaging the circumflex artery. The final frame is counted as the moment the dye first reaches the distal landmark branch, without requiring complete opacification. Usually, the last frame is most accurately determined by advancing the cine film past the initial opacification of the endpoint branch, and then reversing frame by frame until the branch is no longer visible [6].

The analysis uses specific distal landmark branches, including the distal bifurcation of the LAD, often called the “mustache.” In the circumflex system, we focus on the distal bifurcation of the segment with the greatest total length that includes the culprit lesion. For the right coronary artery (RCA), attention is given to the first branch of the posterolateral artery. Notably, the LAD’s frame count is much higher. To address this difference in distance to distal arterial landmarks, we adjust the LAD TIMI frame count by dividing it by 1.7 [6]. Different references suggest CTFC cutoff values of 21 or 27 [6] to indicate normal flow; in this study, we used 27.

One expert interventionalist, blinded to the peak glycemia and patients’ diabetes history, performed an offline analysis and reported the TIMI, CTFC, and SYNTAX scores. The SYNTAX score was calculated using the following online calculator: https://syntaxscore.org/calculator/syntaxscore/frameset.htm.

Bedside echocardiography was performed within the first 24 h after PPCI using the GE Vivid S60N to evaluate cardiac function and structure. Left ventricular function was estimated visually by assessing LVEF. The chamber size [7], valvular diseases [8], and diastolic function [9] were assessed according to the latest guidelines.

### 2.3. Statistical Analysis

Data are presented as mean ± SD or frequency. The study aimed to analyze the relationship between various clinical factors and the occurrence of no‐reflow in patients undergoing PPCI, using SPSS for data analysis. The normal flow and no‐reflow groups were compared with an unpaired *t*‐test and Fisher’s exact test. Logistic regression was also employed for multifactorial predictors. A *p* value less than 0.05 was considered statistically significant. Analyses were conducted using SPSS Version 26.

## 3. Result

The study enrolled 252 patients, with 81.7% being male. Participants were divided into two groups based on the TIMI flow grade after PPCI. Table 1 shows the detailed demographic and laboratory data. The no‐reflow group included 107 participants (42.4%) with flow Grades 0–2, while the normal flow group had 145 participants (57.6%) with TIMI flow Grade 3. There were no significant differences in demographic factors like age and gender between the no‐reflow and normal flow groups, according to the available data. However, the prevalence of smoking was significantly higher in the no‐reflow group (55.14% vs. 30.34%).

**TABLE 1:** Comparison of demographic data, risk factors, and laboratory results between the normal flow group and the no‐reflow group.

	**Normal flow (*n* = 145)**	**No-reflow (*n* = 107)**	**p** **value**
*Demographic data*			
Age	60.3 ± 10.9	60.4 ± 10.3	0.925
Gender			
Male	120 (82.76%)	86 (80.37%)	0.628
Height (cm)	171.7 ± 6.0	171.8 ± 6.7	0.839
Weight (kg)	74.9 ± 7.2	76.0 ± 7.5	0.246
Body surface area (m^2^)	1.87 ± 0.11	1.89 ± 0.12	0.392
Body mass index (kg/m^2^)	25.4 ± 1.8	25.7 ± 1.7	0.159
Systolic blood pressure (mmHg)	128.4 ± 7.2	127.8 ± 7.5	0.495
Diastolic blood pressure (mmHg)	79.5 ± 5.5	79.4 ± 5.6	0.842
Heart rate (beat per minute)	82.2 ± 5.7	82.0 ± 5.4	0.700
Family history of coronary artery disease	12 (8.28%)	6 (5.6%)	0.416
Cigarette	44 (30.34%)	59 (55.14%)	0.001
Alcohol	2	2	0.758
Diabetes	24 (16.55%)	40 (37.38%)	0.001
Hypertension	74 (51.0%)	66 (61.7%)	0.093
Hyperlipidemia	47 (32.41%)	60 (56.07%)	0.001
Hx of known coronary artery disease	12 (8.28%)	29 (27.10%)	0.001
Hx of cerebral vascular accident	4 (2.8%)	3 (2.8%)	0.983
Hx of chronic kidney disease	6 (4.1%)	3 (2.8%)	0.573
Hx of obstructive sleep apnea	0	1	0.243
Peripheral artery disease	1	2	0.393

Drug Hx			
ASA	10 (6.9)	32 (29.9)	0.001
Clopidogrel	10 (4.8)	2 (1.9)	0.211
ACEi/ARB	32 (22.1)	19 (17.8)	0.400
Aldactone	6 (6.2)	3 (2.8)	0.573
B‐blocker	9 (6.2)	12 (11.2)	0.155
Statin	20 (13.8)	19 (17.8)	0.390
Diuretic	10 (6.9)	9 (8.4)	0.653
Oral antidiabetes	28 (19.3)	42 (39.3)	0.001
Insulin	6 (4.1)	11 (10.3)	0.055
Lab data			
Peak glycemia (mg/dL)	179.4	220.6	0.001
HbA1c^∗^ (%)	5.6 ± 0.8	6.2 ± 1.6	0.001
CRP (mg/dL)	10.3 ± 23.3	16.2 ± 32.0	0.088
Uric acid (mg/dL)	4.1 ± 1.3	4.5 ± 1.3	0.031
WBC (10^3^/mm^3^)	10.4 ± 2.7	9.9 ± 3.2	0.217
HB (g/dL)	13.3 ± 1.7	13.5 ± 1.7	0.382
PLT (10^3^/mm^3^)	199.5 ± 71.4	199.1 ± 45.8	0.961
AST (U/L)	58.0 ± 121.8	84.0 ± 123.6	0.097
ALT(U/L)	37.2 ± 72.4	40.8 ± 72.4	0.630
ALP(U/L)	184.3 ± 37.8	191.6 ± 57.9	0.226
Urea (mg/dL)	32.4 ± 17.6	35.3 ± 21.8	0.245
Cr (mg/dL)	1.0 ± 0.3	1.0 ± 0.2	0.324
LDL^∗^ (mg/dL)	95.4 ± 13.5	104.4 ± 22.6	0.001
HDL^∗^ (mg/dL)	38.4 ± 7.6	40.5 ± 7.4	0.026
TG^∗^ (mg/dL)	103.6 ± 21.4	103.6 ± 19.7	0.978
Chol^∗^ (mg/dL)	159.9 ± 22.8	163.6 ± 21.3	0.192
PT(s)	13.0 ± 1.5	13.0 ± 1.7	0.941
INR	1.1 ± 0.2	1.1 ± 0.3	0.988
PTT (s)	30.6 ± 11.4	31.2 ± 17.9	0.773

^∗^Result of the laboratory data on the first day after PPCI in the fasting state.

Furthermore, a larger proportion of patients in the no‐reflow group had comorbidities such as diabetes (37.38%), hyperlipidemia (56.07%), and a history of CAD (27.10%) compared to the normal flow group (16.55%, 32.41%, and 8.28%, respectively). Both admission HbA1c (6.1% vs. 5.6%) and in‐hospital peak glycemia levels (220.6 vs. 179.4 mg/dL) were significantly higher in patients with no‐reflow than in those with normal reflow. This significant difference extended to other laboratory parameters, where patients with no‐reflow consistently showed higher values than those with normal reflow. These include uric acid (4.5 vs. 4.1 mg/dL, *p* = 0.031), HDL (40.5 vs. 38.4 mg/dL, *p* = 0.026), and LDL levels (104.4 vs. 95.4 mg/dL, *p* < 0.001). Although patients with no‐reflow had higher CRP levels, the difference was not statistically significant (*p* value = 0.088).

Regarding echocardiographic parameters, lower LVEF, RV dysfunction, and RV enlargement were significantly more common in the no‐reflow group. There was not enough statistical evidence of reduced LV enlargement in the no‐reflow group, but it was less frequent in normal‐flow patients (Table 2).

**TABLE 2:** Comparison of echocardiographic features in the normal flow and no‐reflow groups.

		**Normal flow (CTFC < 27)**	**No-reflow (CTFC ≥ 27)**	**p** **value**

LVEF		40.8	37.29	0.004

LV systolic dysfunction	Normal and preserved function	20 (13.8)	7 (6.5)	0.040
	Mild	61 (42.1)	37 (34.6)	
	Moderate	52 (35.9)	45 (42.1)	
	Severe	12 (8.3)	18 (16.8)	

LV enlargement	Mild	2	4	0.726
	Moderate	1	0	
	Severe	1	0	

LVH	Mild	14	10	0.271
	Moderate	0	4	
	Severe	1	2	

RV dysfunction	Mild and moderate	4 (2.8)	15 (14.0)	0.001

RV enlargement	Mild and Moderate	0 (0)	6 (5.6)	0.005

sPAP (mmHg)		26.5 ± 3.4	26.7 ± 3.8	0.713

MR (mild, moderate)		135	93	0.180

TR (mild, moderate)		113	90	0.470

**FIGURE 1:**
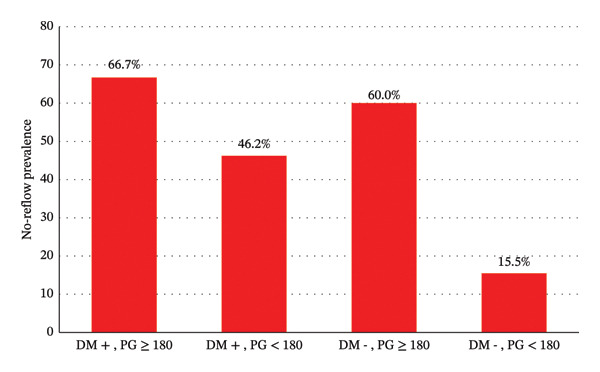
Distribution of the no‐reflow phenomenon based on the presence of diabetes and peak glycemia (PG) level.

The angiographic characteristics (Table 3) showed significant differences between the two groups in the number of narrowed vessels and the SYNTAX score. Notably, the no‐reflow group had a significantly higher prevalence of multivessel disease (63.1%) compared to the normal flow group (44.1%) (*p*‐value = 0.001). The distribution of infarction‐related arteries also differed significantly between the normal and no‐reflow groups (*p* value = 0.003). The LAD was involved in 57% of the no‐reflow group and 36.6% of the normal flow group. The involvement of the RCA as the target lesion was more common in the normal reflow group than in the no‐reflow group (16.6% vs. 7.5%, respectively). Figure 1 illustrates the prevalence of no‐reflow considering the presence of diabetes and peak glycemia levels (below or above 180 mg/dL).

**TABLE 3:** Comparison of angiographic findings between the normal flow group and the no‐reflow group.

	**Normal flow (CTFC < 27)**	**No-reflow (CTFC ≥ 27)**	**p** **value**

Number of narrowed vessels			0.001
Single vessel disease	81 (55.9)	39 (36.4)	
Two vessel disease	37 (25.5)	27 (25.2)	
Three vessel disease/left main disease	26 (18.6)	39 (38.3)	
Culprit lesion			0.003
LAD	53 (36.6)	61 (57.0)	
LCx	24 (16.6)	8 (7.5)	
RCA	68 (46.8)	38 (35.5)	
CFTC	20.6 ± 5.4 (8–26)	43.4 ± 12.5 (28–66)	0.001
SYNTAX	15.7 ± 8.3	23.7 ± 9.9	0.001

Based on logistic regression, no interaction was observed between diabetes mellitus and peak glycemia levels above 180 mg (OR = 0.286, 95% CI = 0.070–1.177, *p* = 0.083). A peak glycemia level of ≥ 180 independently increased the odds of exhibiting the no‐reflow phenomenon (OR = 8.16, 95% CI: 4.1–16.2, *p* ≤ 0.001) (Table 4).

**TABLE 4:** The odds ratio of no‐flow occurrence based on the presence or absence of diabetes and peak glycemia levels.

	OR	95% CIs	*p* value
No DM and PG < 180 mg/dL (reference)	1	—	—
DM and PG < 180 mg/dL	4.661	1.385–15.689	0.013
No DM and peak glycemia ≥ 180 mg/dL	8.156	4.102–16.219	< 0.001
DM and peak glycemia ≥ 180 mg/dL	10.875	4.938–23.948	< 0.001

## 4. Discussion

This study investigated the association of the no‐reflow phenomenon in patients with STEMI undergoing direct PCI with peak glycemia. The findings indicated a significant association between the occurrence of the no‐reflow phenomenon and factors such as a history of diabetes, peak glycemia, a history of cigarette smoking, a history of CAD, and higher LDL levels in both diabetic and nondiabetic patients.

The no‐reflow phenomenon mainly occurs due to distal embolization and reperfusion injury. Patients may experience chest pain along with an elevation in the ST segment on the electrocardiogram [10, 11]. Coronary blood perfusion is a key clinical indicator for assessing the success of PCI in patients with STEMI. Restoring microvascular function and achieving successful reperfusion influence both functional and clinical outcomes after an AMI [12–14]. Therefore, recent studies focus on identifying predictors of the no‐reflow phenomenon in patients with STEMI before PPCI. Research shows that both hyperglycemia and peak glycemia are associated with the highest mortality rates, regardless of whether patients have diabetes [15, 16]. Hyperglycemia is strongly linked to AMI and correlates with high mortality rates in diabetic patients following an AMI [17, 18]. In this study, effective glycemic control may be a helpful strategy for preventing no‐reflow, regardless of diabetic status.

Additionally, no‐reflow has been associated with higher cholesterol levels and increased thrombus burden [19, 20]. Our study also notes that elevated LDL cholesterol in the no‐reflow group emphasizes the importance of this risk factor. This research does not assess HDL levels as they are highly affected in post‐STEMI states.

The hemodynamic vulnerability of hyperglycemic STEMI patients was reported in a retrospective study that assessed the predictive value of baseline characteristics, angiographic, echocardiographic, and laboratory data for in‐hospital mortality in 319 STEMI patients with cardiogenic shock who underwent PPCI. In the multivariate analysis, chronic renal failure, post‐PCI TIMI flow ≤ 2, plasma glucose and lactate level, blood urea nitrogen level, tricuspid annular plane systolic excursion (TAPSE), and LVEF were independent predictors of in‐hospital mortality. In this retrospective, only the blood glucose level at admission was measured, and the CTFC was not assessed [21]. Another retrospective study found an association between LVEF, chronic renal failure, post‐PCI TIMI flow ≤ 2, plasma glucose and lactate levels, and mortality among ACS patients with intra‐aortic balloon pump support in the intensive coronary care unit [22]. Again, serial measurement of the blood glucose and CTFC was not evaluated in their study.

Multiple prognostic scoring systems already incorporate glucose components, underscoring glycemia as a direct determinant of adverse outcomes in MI. In one study, the predictive performance of the “Intermountain Risk Score” (IMRS) and the “SYNTAX Score II” was compared. The IMRS combines variables from the CBC and metabolic panel, including sodium, potassium, chloride, bicarbonate, blood urea nitrogen, creatinine, glucose, and calcium levels. The IMRS outperformed the SYNTAX Score II in terms of predicting both short‐ and long‐term mortality [23].

The exact mechanism by which hyperglycemia influences the occurrence of no‐reflow during PCI remains unclear. Multiple potential pathological pathways have been suggested to explain the link between hyperglycemia and the no‐reflow phenomenon. First, hyperglycemia increases levels of intercellular adhesion molecule‐1 (ICAM‐1) and P‐selectin, which promote leukocyte adhesion to capillaries, thereby increasing capillary bed obstruction [24]. Furthermore, hyperglycemia contributes to the formation of additional microthrombi, a significant factor in the no‐reflow phenomenon. Microemboli generated during PPCI procedures can cause microvascular dysfunction. The underlying process involves microvascular plugging due to platelet and neutrophil activity, increased platelet activation, a large thrombus burden, reperfusion injury, ischemic injury, endothelial dysfunction, inflammation, oxidative stress, interstitial edema, and swelling of myocardial cells. Hyperglycemia may amplify these mechanisms, thereby worsening no‐reflow [25]. The role of hyperglycemia in activating blood coagulation has also been investigated in prior studies. Acute fluctuations in blood glucose appear to be associated with changes in the coagulation cascade, which may promote thrombosis [26, 27]. Stress hyperglycemia in patients with acute MI is a transient response triggered by the initial release of catecholamines, cytokines, and cortisol, rather than simply reflecting the patient’s underlying glucose metabolism. Deckers et al. [28] examined a large cohort of 11,324 patients, of whom 41% had blood glucose levels > 140 mg/dL at admission [28]. Stress hyperglycemia reduces fibrinogen half‐life and increases fibrinopeptide A levels, as well as prothrombin fragments, Factor VII, and platelet aggregation [29, 30]. Collectively, these findings indicate increased thrombotic activity.

It is important to acknowledge certain limitations of the current study. The cross‐sectional design should be noted, but it is worth emphasizing that our cohort was relatively large and included consecutive patients. Since the study focused on patients with STEMI undergoing PPCI, the results may not apply to all ACS patients. Additionally, we recognize the potential for residual confounding from unmeasured variables that could influence outcomes.

## 5. Conclusion

**FIGURE 2:**
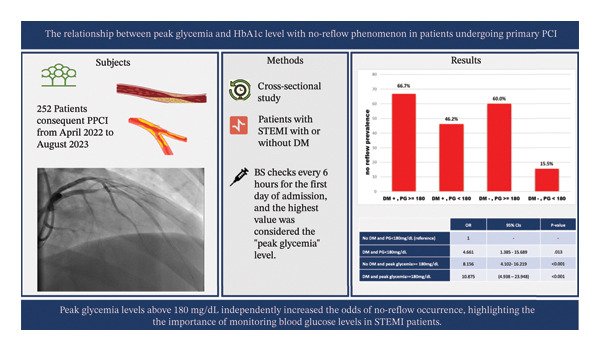
Summary of the research process and findings.

In this study, the group of patients who experienced no‐reflow had higher LDL levels, a greater prevalence of diabetes mellitus, and a history of smoking. High peak glycemia increases the likelihood of experiencing the no‐reflow phenomenon in patients with STEMI undergoing PPCI (Figure 2). Blood glucose levels should be closely monitored in all patients with STEMI, regardless of their diabetic status.

## Author Contributions

Babak Geraiely: conceptualization, project administration, data curation, supervision, and methodology.

Maryam Mehrpooya: investigation, supervision, and methodology.

Golrokh Ghaffari: resources, project administration, data curation, and methodology.

Mohsen Faghihinia: resources and data curation.

Farnoosh Larti: supervision, methodology, and review and editing of the original and final draft.

Elnaz Shahmohamadi: writing the original draft and methodology.

## Funding

The authors have nothing to report.

## Ethics Statement

The Research Ethics Committee of Tehran University of Medical Sciences (IR.TUMS.MEDICINE.REC.1401.256) approved the study protocol. All treatment methods were conducted in accordance with relevant guidelines and regulations.

## Consent

Written informed consent was obtained from the study population.

## Conflicts of Interest

The authors declare no conflicts of interest.

## Data Availability

The data supporting this study’s results can be provided upon reasonable request from the corresponding authors.
